# Microbial community succession of home aquarium biofilters associated with early establishment of comammox *Nitrospira*

**DOI:** 10.1093/ismeco/ycaf212

**Published:** 2025-11-14

**Authors:** Michelle M McKnight, Natasha Szabolcs, Alyssa Graham, Josh D Neufeld

**Affiliations:** Department of Biology, University of Waterloo, Waterloo, ON N2L 3G1, Canada; Department of Biology, University of Waterloo, Waterloo, ON N2L 3G1, Canada; Department of Biology, University of Waterloo, Waterloo, ON N2L 3G1, Canada; Department of Biology, University of Waterloo, Waterloo, ON N2L 3G1, Canada

**Keywords:** aquarium, biofilter, nitrification, 16S rRNA gene sequencing, microbial community succession, metagenomics

## Abstract

Nitrification in aquarium biofilters transforms toxic ammonia (NH₃/NH₄^+^) into less toxic nitrate (NO₃^-^) via nitrite (NO₂^-^). Known freshwater aquarium nitrifiers include ammonia- and nitrite-oxidizing bacteria, ammonia-oxidizing archaea (AOA), and complete ammonia-oxidizing *Nitrospira* (CMX), with CMX recently shown to dominate most freshwater aquarium biofilters. However, little is known about nitrifier succession during aquarium establishment in home settings. Based on CMX prevalence in mature aquariums and the rapid growth of ammonia-oxidizing bacteria (AOB), we hypothesized that AOB initially dominate before CMX establish. To test this, we monitored microbial succession and water chemistry in three home aquariums over 12 weeks, collecting weekly samples from aquarium water, biofilter beads, and sponge filters. Biofilter DNA was analyzed via 16S rRNA gene sequencing and quantitative PCR (qPCR) targeting *amoA* genes. Nitrification reduced ammonia and nitrite to undetectable levels by week 3 in two aquariums and by week 8 in the third. Ammonia oxidizer detection by qPCR coincided with the onset of ammonia oxidation, with AOA preferentially colonizing biofilter beads. Metagenomic profiling of week 12 biofilter samples confirmed AOA and comammox *Nitrospira amoA* genes in all aquariums, along with *nxrB* genes from both comammox and canonical *Nitrospira* nitrite oxidizers. These results provide insight into the establishment of ammonia oxidizers in residential aquariums. Future work should explore factors influencing nitrifier community assembly, including inoculation sources (e.g. live plants, biological supplements), fish load, and water chemistry.

## Introduction

When starting a new aquarium, it is essential to promote establishment of a microbial community within the associated biofilter that conducts nitrification. Nitrification is an aerobic respiratory process that converts relatively toxic ammonia (NH_3_/NH_4_^+^) waste via the intermediate nitrite (NO_2_^-^) to less toxic nitrate (NO_3_^-^). This process is critical for closed aquatic systems, such as home aquariums and recirculating aquaculture systems (RAS), because ammonia released as nitrogenous waste from fish or through organic matter decomposition can accumulate to harmful levels (e.g. > 0.1 mg/L NH_3_-N), negatively impacting resident aquatic life.

Nitrification can occur in two discrete biological steps, with ammonia-oxidizing bacteria (AOB; e.g. *Nitrosomonas* spp.) or archaea (ammonia-oxidizing archaea [AOA]; *Nitrosotenuis* spp.) performing the initial oxidation of ammonia to nitrite, followed by further oxidization of nitrite to nitrate by nitrite-oxidizing bacteria (NOB; e.g. *Nitrospira* spp.). In contrast, complete AOB or “comammox” (CMX) bacteria, are capable of oxidizing ammonia through to nitrate. Complete ammonia oxidation may be favorable in aquarium environments because it circumvents the release of nitrite into the environment, which can also be toxic to aquatic organisms [1]. Prior to the discovery of AOA and comammox *Nitrospira* in 2005 and 2015 [2–4], respectively, AOB were considered by the aquarium industry as dominant ammonia-oxidizers, with AOB supplements sold for use by aquarists to expedite nitrogen cycling in new aquariums [5, 6]. However, recent studies reassessing the presence of all three groups of ammonia oxidizers (AOB, AOA, and CMX) have revealed that comammox *Nitrospira* and AOA are among the dominant ammonia oxidizers in established freshwater aquarium biofilters, with AOB and AOA dominant in saltwater biofilters in the absence of comammox *Nitrospira* [7, 8]. With a revised understanding of the ammonia oxidizers involved in aquarium nitrification, there is still a need to explore the dynamics of microbial community members during biofilter establishment, including all three potential groups of ammonia oxidizers that often coexist in aquarium biofilm communities [9–11].

Aquarium biofilters are a type of fixed-film bioreactor, often involving carrier materials like ceramic beads and polyurethane sponges that support biofilm growth, with flow rates to help control biofilm thickness and ensure sufficient oxygen and nutrient transfer into the biofilm [12]. In a newly established aquarium, biofilter nitrifier communities accumulate in response to the availability of ammonia and nitrite, with the initial growth of ammonia oxidizers in the biofilm, followed by an increase in NOB in response to the nitrite production of strict ammonia oxidizers. The result of a well-established nitrifying community is to maintain a low-level of total ammonia nitrogen (TAN; NH_3_/NH_4_^+^), with undetectable levels of nitrite, and accumulating levels of nitrate that can be lowered via water changes or uptake by live plants.

Although the establishment of nitrifiers in aquarium biofilters can be accelerated by seeding with nitrifiers (AOB; *Nitrosomonas* and NOB; *Nitrobacter*) [13], nitrifying communities also develop in biofilters without supplementation, suggesting that microbial nitrifiers are introduced to the aquarium through drinking water systems, fish, plants, and other environmental sources [14, 15]. However, no research has explored whether it is common to have all three groups of ammonia oxidizers naturally introduced into a home aquarium, and only a few studies have explored temporal dynamics of ammonia oxidizers within these systems [16].

Dynamics within biofilter microbial communities are complex and interactions occur not only between autotrophic nitrifiers, but also with the many heterotrophic community members that play an important role in the degradation of organic waste that accumulates in aquarium systems [17]. There is competition for both resources (e.g. oxygen, C and N sources) and space within the biofilm among different autotrophic ammonia oxidizers and heterotrophic bacteria, because the diversity of the microbial community increases during biofilm maturation [18]. Ammonia oxidizers have evolved distinct strategies for niche differentiation depending on environmental conditions. With higher growth rates, AOB are better able to dominate in an environment early but can be displaced by both AOA and comammox *Nitrospira*, which tend to possess higher ammonia affinities and are thus better able to compete for substrate in oligotrophic environments [19, 20]. This pattern of ammonia oxidizer succession was observed in an experimental freshwater aquarium biofilter, where the AOB that had initially established were outcompeted by AOA after a 6-month stabilization period [16]. Currently, it is unclear how comammox *Nitrospira* establish within aquarium biofilters alongside AOA and AOB representatives.

We hypothesized that ammonia oxidizers capable of faster growth (e.g. AOB), with a preference for higher ammonia concentrations, would establish early in time series, and nitrifiers better adapted to lower ammonia niches and with slower growth rates (i.e. AOA, CMX) would establish later in the biofilter sampling period. To test this hypothesis, this study monitored biofilters of three independently established freshwater aquariums, from initial start-up to maturation, evaluating the succession of the biofilm microbial populations, with a focus on nitrifiers.

## Materials and methods

### Home aquarium set-up and sample collection

A home aquarium experiment was conducted to assess ammonia oxidizer abundances in biofilters of newly established freshwater aquariums that were subjected to unregulated feeding and fish stocking schedules. Three individuals participated in this experiment by establishing a new aquarium in their homes from which they collected water and biofilter samples over the first three months (12-weeks) of operation. The sample size of three aquariums is similar to previously published experiments examining perturbation effects on microbial community succession in saltwater aquariums, where two independent aquariums were studied [21]. Participants were provided with a research package that included set-up guidelines, sampling instructions, documentation sheets, and sampling materials, and asked to collect ceramic bead, sponge, and water samples weekly in addition to 24 hours before and 48 hours after the addition of fish (Supplemental methods).

### Water chemistry

Assays were performed to determine the concentrations of total ammonia (NH_3_/NH_4_^+^), nitrite, and nitrate in collected aquarium water samples following previously established protocols (see Supplemental methods). The adapted ortho-phalaldehyde assay was used to determine total ammonia concentration [22, 23], and the Griess reagent was used for nitrite and nitrate quantification [24].

### DNA extraction

Microbial DNA was extracted from biofilter sponge and ceramic beads using the DNeasy PowerSoil Pro Kit (Qiagen, Hilden, Germany) following the manufacturer’s protocol with modifications (Supplemental methods). Samples were run on a 1% agarose gel stained with EtBr to assess DNA quality, followed by quantification using the Qubit dsDNA HS Assay Kit (Thermo Fisher Scientific, Waltham, MA, USA) and an assessment of sample purity using the NanoDrop 2000 (Thermo Fisher Scientific, Waltham, MA, USA). Extracted DNA samples were stored at −20°C until further use.

### Quantitative PCR

To quantify ammonia-oxidizing microorganisms present in biofilter bead and sponge samples, quantitative PCR (qPCR) was performed on all extracted DNA samples (Supplemental methods). Primers targeting the ammonia monooxygenase subunit A gene (*amoA*) were used to quantify the abundances of AOB, AOA, and comammox *Nitrospira* within the biofilter samples following previously established protocols [8]. Additionally, qPCR was performed with the 515F-Y/806R primer set targeting the V4 region of bacterial and archaeal 16S rRNA genes [25, 26]. All qPCR amplifications were run using the CFX Opus 96 Real-Time PCR System (Bio-Rad, Hercules, CA, USA) and analyzed with Bio-Rad CFX Maestro Software (version 2.3), including quantification and melt curve analysis. All generated qPCR standard curves had *R*^2^ values >0.99 and amplification efficiencies ranging from 87–97%. Final qPCR products were confirmed on 1% agarose gels. All quantification results are presented as copies detected in a bead sample (i.e. single ceramic bead), or sponge sample (1 × 1 × 2 cm^3^ in size) used for DNA extraction.

### 16S rRNA gene and metagenomic sequencing

Sequencing of the V4-V5 region of the 16S rRNA gene for all bead and sponge samples was carried out and analyzed using previously established protocols on a MiSeq System (Illumina, San Diego, CA, USA) [8, 27], as described in the Supplemental methods. Additionally, metagenomic sequencing was performed on the final bead and sponge samples collected during the home aquarium experiment (week 12 samples) to evaluate the potential metabolic functions of microbial communities established in the biofilter bead and sponge material after three months (Supplemental methods).

## Results

### Home aquarium sampling

Home aquariums were established using the provided materials and a common municipal tap water for all three residences, with choices regarding fish stock, decoration, feeding, and maintenance left up to personal preference of the participants (Table 1). Once stocked, Aquarium 1 was maintained at ~27°C and contained live plants and seven fish and live plants. Aquarium 2 also contained live plants, but was stocked with 16 fish and was maintained at a water temperature of ~23°C. Aquarium 3 contained artificial plants, along with seven fish, and a water temperature of ~24°C.

**Table 1:** Contents of participating aquariums in home aquarium experiment.

**Aquarium**	**Fish**	**Food**	**Water source/ temperature (°C)**	**Water treatment**	**Accessories**
1	3 Burmese spotted danios; 4 rainbowfish	TetraMin Tropical Flakes (Tetra)	Municipal water/ 27	AquaSafe (Tetra)	Live plants: Java fern, *Anubias*; natural gravel
2	1 *Betta splendens*; 3 Rasboras (week 2); 6 *Corydoras* (week 3); 6 fish (other)	Color Flakes (Cobalt Aquatics)	Municipal water/ 23	Aquarium Conditioner (Marineland)	Gravel; driftwood; rock; live plants
3	3 Rummy-nose tetra; 3 Red-eyed tetra; 1 Chocolate Oranda goldfish	TetraPro Tropical Color Crisps (Tetra); Omega One Freeze Dried Tubifex Worms (Omega One)	Municipal water/ 24	Aqua Plus (Nutrafin); Cycle (Hagen Industries)	Gravel; toy plane; fake pottery; artificial plants

A total of 14 bead and 14 sponge samples were collected from each aquarium (Table S1). To assess the impact of fish addition on the biofilter microbial communities, bead and sponge samples were collected 24 hours before adding fish to the aquarium (pre-fish) and 48 hours after adding fish (post-fish). The following week, bead and sponge samples were collected for “week 1” and subsequently for the following 11 weeks, for a total of 12 weeks of sampling, in addition to the pre- and post-fish sample collections.

### Aquarium nitrification

**Figure 1:**
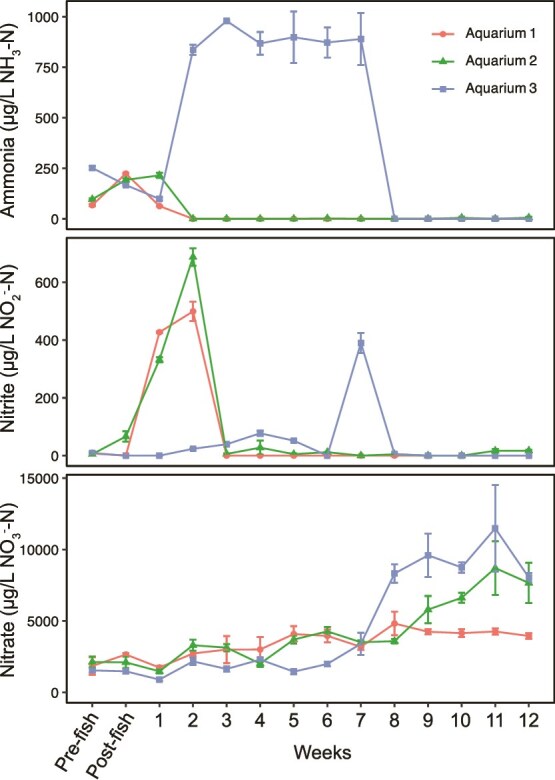
Water chemistry measurements quantifying levels of total ammonia, nitrite, and nitrate as micrograms of nitrogen per liter in aquarium water for all three home aquariums during the first 12-weeks following aquarium start-up. Data points represent the average of technical triplicate measurements for each sample, with error bars representing the standard deviation of replicates. The ammonia measurement represents the total ammonia in the sample, including both ionized and un-ionized forms (total ammonia).

Samples of aquarium water collected during weekly biofilter samplings were used to measure total ammonia, nitrite, and nitrate. Before fish introductions, measurements showed relatively low total ammonia concentrations in Aquarium 1 and 2 samples (Fig. 1), with a higher concentration for Aquarium 3 (~250 μg/L NH_3_-N). At the start, all aquariums had no detectable nitrite and concentrations of nitrate around 2 mg/L NO_3_^-^-N (Fig. 1). The initial elevated concentration of nitrate likely originates from municipal tap water used to fill all three aquariums, which is known to have concentrations ~500 μg/L NO_3_^-^-N in the municipality where participants resided [28].

After fish introductions, aquarium ammonia concentrations increased, associated with food added and fish waste produced. For Aquariums 1 and 2, a relatively small increase in ammonia was observed after fish addition, but ammonia was undetectable in these two aquariums from week 2 onwards (Fig. 1). The maintenance of ammonia concentrations below detection limits and increasing nitrite concentrations reflects active ammonia oxidation activity established during week 1. The concentration of nitrite in Aquariums 1 and 2 reached a maximum during week 2 at concentrations of 499 and 687 μg/L NO_2_^-^-N, respectively, and then decreased below detection limits the following week (Fig. 1). In parallel with nitrite oxidation, nitrate concentrations increased for Aquariums 1 and 2 over the experimental time frame.

In contrast to active nitrification observed in both Aquarium 1 and 2, which both maintained undetectable levels of ammonia from week 2 onwards, Aquarium 3 was not associated with nitrification activity until the second month. Although there was an initial decrease in ammonia concentration for Aquarium 3 between pre-fish and week 1 samples, ammonia increased to >750 μg/L NH_3_-N between week 1 and week 2 and stayed at this level until week 7 (Fig. 1). At week 7, nitrite concentrations rose to ~400 μg/L NO_2_^-^-N from previously undetectable levels, indicative of ammonia oxidation activity in the biofilter (Fig. 1). The following week, both ammonia and nitrite were undetectable, and nitrate concentrations increased, reflecting nitrification activity in Aquarium 3 (Fig. 1).

### Quantification of biofilter ammonia oxidizers with qPCR

**Figure 2:**
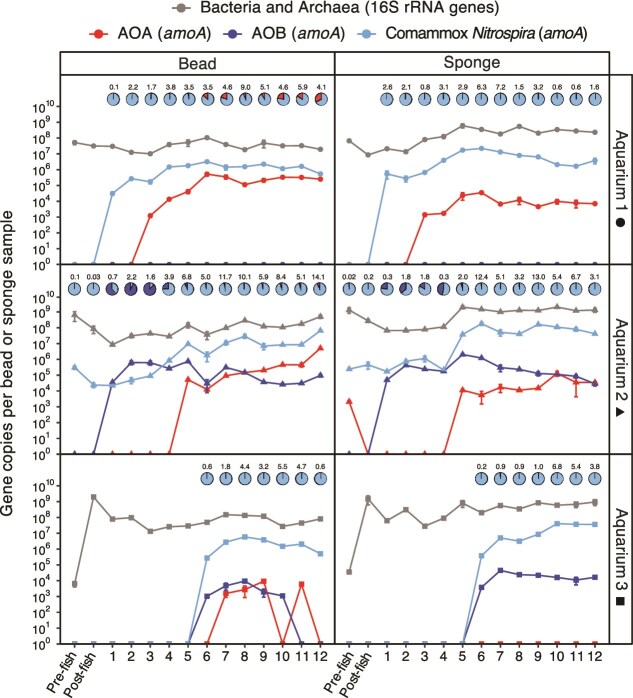
Abundances of 16S rRNA and *amoA* gene copies per bead or per sponge sample for ammonia oxidizers quantified using qPCR. Data points show the detected copies per bead or per sponge sample used for DNA extraction for all four quantified genes: AOA *amoA*, AOB *amoA*, comammox *Nitrospira amoA*, and the V4 region of the 16S rRNA gene. The qPCR for each sample was run as technical duplicates with average values shown and error bars representing the standard deviation of duplicates. Data point shapes correspond to each aquarium, with circle, triangle, and square representing aquarium 1, 2, and 3, respectively. Pie charts represent the relative proportions of ammonia oxidizer groups detected within the sample, and in samples where no *amoA* genes were detected there is no chart displayed. Numbers above pie charts represent the relative abundance (%) of the total community (i.e. 16S rRNA gene copy number) that is represented by ammonia oxidizers (*amoA* gene copy number) for each sample.

To quantify nitrifier community abundances within aquarium biofilters, qPCR was performed with primer sets targeting *amoA* genes from AOA, AOB, and clade A comammox *Nitrospira*. Additionally, the abundance of prokaryotic templates based on 16S rRNA gene copies was quantified using primers targeting the V4 region of the 16S rRNA gene for bacteria and archaea [25, 26]. During the 12-week sampling period, including pre- and post-fish sample collections, the 16S rRNA gene copies detected in both the biofilter bead and sponge samples ranged between 10^6^–10^9^ copies per bead or sponge sample (Fig. 2). The only exception was Aquarium 3 pre-fish samples, where quantities of 16S rRNA genes were only 6.4 × 10^3^ and 3.5 × 10^4^ copies per bead and sponge samples, respectively. These first samples collected from Aquarium 3 also had <5 ng of total DNA extracted from both bead and sponge samples (Table S1). However, for post-fish samples, these 16S rRNA gene abundances were in the same range as Aquariums 1 and 2.

The succession of ammonia-oxidizing groups (AOA, AOB, and comammox *Nitrospira*) was distinct for each aquarium. Aquarium 1 had detectable clade A comammox *Nitrospira amoA* gene copies as early as week 1 for both bead and sponge samples (Fig. 2). Comammox *Nitrospira amoA* genes remained elevated at magnitudes of 10^5^–10^6^ copies per bead and at higher levels of 10^5^–10^7^ copies per sponge sample through to week 12. In contrast, AOB *amoA* genes were below the detection limit for both bead and sponge throughout weeks 1–12, including pre- and post- fish sampling (Fig. 2). Although at a lower abundance than comammox *Nitrospira amoA* genes, AOA *amoA* genes were detectable in both Aquarium 1 bead and sponge from week 3–12, with copy numbers 10–100 fold greater per bead than per sponge sample, suggesting a preference for growth in biofilter beads (Fig. 2). First detectable in week 3, AOA *amoA* genes increased to magnitudes of 10^5^ copies per bead from weeks 6–12. By the final sampling at week 12, AOA represented more than a quarter of all *amoA* genes detected in biofilter beads.

For the Aquarium 2 biofilter, comammox *Nitrospira amoA* genes were detected in pre-fish samples at a magnitude of 10^5^ copies per bead or sponge sample, suggesting that they were introduced during establishment of the aquarium prior to fish addition (Fig. 2). By week 5, comammox *Nitrospira amoA* genes increased to concentrations of 10^6^ copies and 10^7^ copies per biofilter bead and sponge sample, respectively. During weeks 7, 8, and 12, comammox *Nitrospira amoA* genes reached their highest at concentrations above 10^7^ copies per bead, while during weeks 6, 9, and 10, magnitudes of 10^9^ copies per sponge samples were detected. The AOA *amoA* genes were not present in both bead and sponge until week 5, despite an initial detection of ~10^3^ copies per sponge sample, suggesting inoculation of AOA into the aquarium during set up (Fig. 2). Similar to Aquarium 1, AOA *amoA* genes in the Aquarium 2 biofilter beads were detected at magnitudes of 10^5^ copies per bead from weeks 8–11, increasing to 10^6^ copies per bead in week 12. Although AOA again showed a preference for bead over sponge biofilter material, the discrepancy in magnitude between sample types was not as great as was observed in Aquarium 1 (Fig. 2). Here, AOA *amoA* genes were detected at magnitudes of 10^4^ copies per sponge sample from weeks 7–12, apart from the 10^5^ copies per sponge sample detected in week 10. In contrast to Aquarium 1, Aquarium 2 had AOB *amoA* genes detected in both biofilter bead and sponge from week 1 onwards. The AOB *amoA* genes reached maximum copies per bead and sponge samples during week 5, representing >80% of the total ammonia oxidizers in the biofilter beads during weeks 2–3 (Fig. 2). In the following weeks, AOB *amoA* gene concentrations decreased to 10^3^ copies per sponge sample by week 12.

Ammonia oxidizer establishment was delayed for the Aquarium 3 biofilter. Comammox *Nitrospira* were not detected until week 6 sampling, reaching 10^6^ copies per bead in the following weeks, and up to 10^7^ copies per sponge sample (Fig. 2). The AOA *amoA* genes were not detectable in Aquarium 3 sponge samples throughout all sample time points (Fig. 2). In contrast, AOB *amoA* genes were detected in biofilter beads during weeks 6–10 at magnitudes of 10^3^ copies per bead, whereas in sponge they were detected at 10^3^ copies per sponge sample during week 6 and increased to 10^4^ copies per sponge sample for the rest of the experiment (Fig. 2).

The proportion of the total prokaryotic community represented by ammonia oxidizers changed throughout the 12-weeks of sampling, making up 1.6%, 3.1%, and 3.8% of prokaryotes in biofilter sponge by week 12 in Aquariums 1, 2, and 3, respectively (Fig. 2). This proportion was even higher in week 12 for the biofilter beads of Aquarium 1 (4.1%) and Aquarium 2 (14.1%), revealing that ammonia oxidizers comprised a higher proportion of the prokaryotic community in biofilter beads than in sponge. However, the relative abundance of ammonia oxidizers was low for Aquarium 3 biofilter beads (0.6%), despite having a similar total DNA yield to that of the Aquarium 1 bead sample (Table S1), suggesting that there was not a well-established group of ammonia oxidizers in the beads or that heterotrophic bacteria were more dominant. Ammonia oxidizers were relatively abundant in the sponge of Aquarium 3, making up 3.8% of the total prokaryotic community with a DNA yield from the sponge sample greater than that of Aquarium 1 and less than that of Aquarium 2 (Fig. 2, Table S1).

Despite individual aquarium variations in ammonia oxidizer proportions, bead and sponge samples from all three biofilters were dominated by comammox *Nitrospira* from weeks 6–12 (Fig. 2). Within the biofilter beads, both comammox *Nitrospira* and AOA were prevalent ammonia oxidizers, although the sponge was dominated solely by comammox *Nitrospira* by week 12. These results suggest that AOA associate primarily with bead surfaces for colonization. The results also indicate that ammonia oxidation activity coincided with the detection of ammonia oxidizers in each aquarium biofilter. Nitrite concentrations of Aquariums 1 and 2 increased between post-fish and week 1 samples, coinciding with the detection of comammox *Nitrospira* in Aquarium 1 and both comammox *Nitrospira* and AOB in Aquarium 2 in sampled biofilter bead and sponge. The delayed start of ammonia oxidation in Aquarium 3, evident from weeks 6 and 7, aligned with the first detection of comammox *Nitrospira* in bead and sponge samples at week 6. The complementarity of increasing nitrite, alongside ammonia oxidizer detection, provides evidence linking biofilter nitrification activity with the nitrifiers detected in biofilter samples.

### Microbial community establishment in the aquarium biofilters

**Figure 3:**
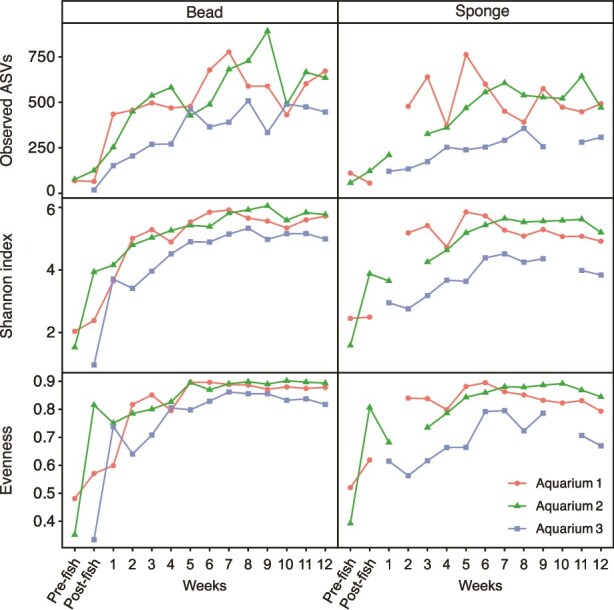
Alpha diversity of microbial communities from all aquarium samples across all 14 sampling time points. Alpha diversity values were determined using observed ASVs for richness, the Shannon index for evenness and richness and Pielou’s evenness index. Six samples had read counts below the rarefaction depth of 9384 and as a result were removed from the analysis. Omitted samples do not have corresponding evenness, Shannon index, or observed ASV values displayed above.

To analyse the total microbial community composition of aquarium biofilter beads and sponge over the 12-week sampling period, 16S rRNA gene sequencing was performed for each aquarium. A total of 10 645 ASVs were identified in the dataset, with an average of 24 277 reads per sample (SD = 11 822 reads). During the first few weeks, both the number of detected ASVs (richness), evenness, and Shannon index values for the biofilter bead and sponge samples increased (Fig. 3). Alpha diversity measures were consistent for week 7–12 samples from each aquarium. By week 12, there were similar numbers of ASVs in Aquarium 1 and Aquarium 2 for the bead and sponge samples, respectively, with higher diversity values for bead samples overall (Fig. 3). Aquarium 3 had lower diversity values for all time points.

**Figure 4:**
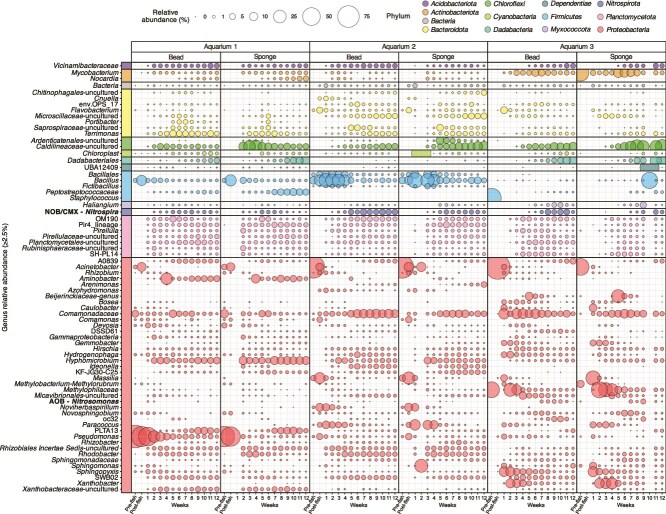
Relative abundance of genera across bead and sponge biofilter samples. Bubble size and numbers indicate the relative abundance of each genus within a sample, with colors representing phylum associations. All genera shown on the plot are present in at least one sample at a relative abundance of 2.5% or greater.

Biofilter microbial community composition during pre-fish, post-fish, and week 1 sampling included *Proteobacteria* and *Firmicutes* in bead and sponge samples for all aquariums (Fig. 4). From weeks 2–12, genera predominantly from the *Proteobacteria* phylum were detected, along with those associated with *Bacteroidota*, *Planctomycetota*, and *Firmicutes*. At the genus level, many similar genera were detected across all three aquariums in bead and sponge biofilters, including *Bacillus*, *Comamonadaceae* spp., *Hyphomicrobium*, *Caldilineaceae* spp., and *Mycobacterium* (Fig. 4). *Nitrospira* spp. were also detected for all aquarium bead and sponge samples, corresponding similarly to qPCR results. Differences in individual aquarium biofilter communities included a higher relative abundance of *Bacillus* observed in Aquarium 2 when compared with Aquariums 1 and 3, and a higher relative abundance of *Mycobacterium* in Aquarium 3 compared to the other two (Fig. 4).

**Figure 5:**
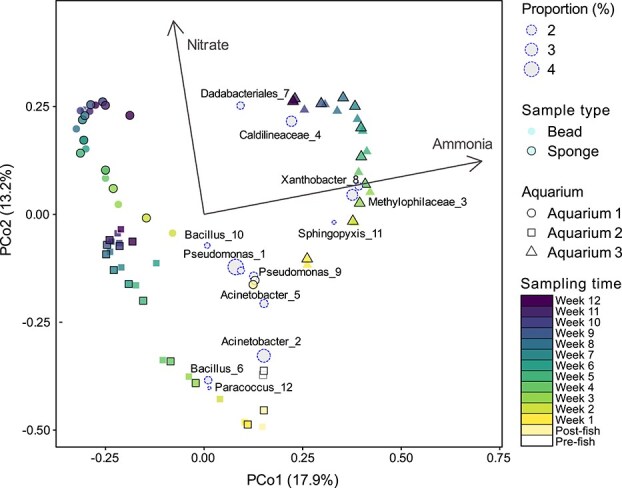
Triplot analysis of the 16S rRNA gene sequence data displays the principal coordinate values of each bead and sponge sample, calculated based on the Bray-Curtis dissimilarity metric for samples rarefied to 9384 reads (samples below this were removed). Variation explained by principal coordinates 1 and 2 are displayed on the *x* and *y* axes, respectively. Dashed circles for ASVs represent average relative abundance values greater than 1% and the location within ordination space reflects relative abundance correlations with microbial community profiles. Arrows represent the direction of significant correlations of environmental variables with beta diversity values above a threshold for inclusion (*R*^2^ *>* 0.2, *P* < .01).

From week 3 onwards, the microbial community profiles of all bead and sponge samples showed clear differences among aquarium biofilters (Fig. 5). Although the unique composition of biofilter communities for Aquariums 1, 2, and 3 were strongest for weeks 9–12 samples, overall beta diversity among aquariums was significantly different across the entire sampling period (PERMANOVA; *R*^2^ = 0.27, *P* = .001). Small but significant differences in microbial community composition were also observed between bead and sponge biofilter samples (PERMANOVA; *R*^2^ = 0.03, *P* = .002; Fig. 5). However, there was no significant interaction between biofilter material type (e.g. bead and sponge) and aquarium (two-way PERMANOA; interaction *R*^2^ = 0.03, *P* = .106), indicating that differences between bead and sponge filters were consistent across aquariums. There was a significant correlation of ammonia concentration to community composition (*R*^2^ = 0.51, *P* = .001), with the vector pointing toward samples from Aquarium 3 with the highest ammonia concentrations (Fig. 5). Beta diversity was also significantly correlated with nitrate concentration, where higher nitrate concentrations were associated with later sampling time points (*R*^2^ = 0.20, *P* = .001; Fig. 5). This was expected because nitrate concentrations increased during the 12-weeks in relation to the nitrification activity that occurred for all three aquariums from week 7 onwards. Triplot analysis revealed specific taxa correlated with the microbial community composition, including *Bacillus* spp. with Aquarium 1 (ASV 10) and Aquarium 2 (ASV 6), and *Caldilineaceae* (ASV 4), *Xanthobacter* (ASV 8), *Methylophilaceae* (ASV 3), and *Sphingopyxis* (ASV 11) with Aquarium 3 (Fig. 5). Despite distinct microbial community compositions of each aquarium, they all showed similar patterns from week 7 onwards, with decreased dissimilarity between samples for each aquarium, and likewise corresponding to a plateau in alpha diversity metrics for that same timeframe (Fig. 3, Fig. 5).

**Figure 6:**
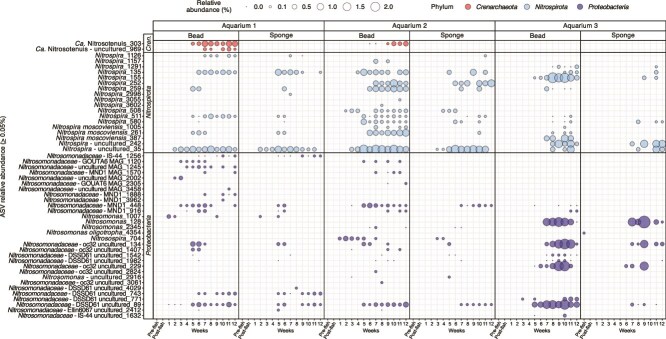
Relative abundance of nitrifier-associated ASVs in bead and sponge microbial communities. Bubbles on the plot display the relative abundance (%) of each ASV associated with nitrification in the microbial community of each biofilter bead and sponge sample. The ASVs were filtered based on having the presence of “nitro” at the beginning of taxonomic rank names. Relative abundance bubbles labeled “0” have a percentage value greater than 0 but equal or less than 0.05%. The absence of a bubble indicates 0% relative abundance of the ASV.

Along with a qPCR assessment of ammonia oxidizer gene abundances, the RA of nitrifier ASVs was also explored. Overall, 16S rRNA gene profiles were consistent with the detection of ammonia-oxidizing microorganisms quantified by qPCR analysis. In biofilter beads from Aquariums 1 and 2, AOA ASVs of *Ca.* Nitrosotenuis were detected at a relative abundance up to ~1% starting from week 5 in Aquarium 1 and week 6 for Aquarium 2 (Fig. 6). Additionally, two unique *Ca.* Nitrosotenuis ASVs were detected in Aquarium 1, with only one AOA-associated ASV detected in Aquarium 2. There were many different ASVs associated with *Nitrospira* genera detected across all aquarium biofilter bead and sponge samples, which appeared at the same time points as qPCR detection (Fig. 5). Multiple *Nitrospira* ASVs were present at most sampling time points and several *Nitrospira* were detected for all three aquariums, including ASVs 35, 135, 259, and 580 (Fig. 5). Although several of these *Nitrospira* ASVs represent comammox *Nitrospira*, others likely represent canonical NOB *Nitrospira*; however, 16S rRNA gene sequences alone cannot be used to determine whether ASVs correspond to comammox *Nitrospira* or canonical NOB [10]. Aside from *Nitrospira*, no other NOB-associated genera were detected in any of the biofilter samples. Several AOB ASVs were detected, including five *Nitrosomonas* ASVs and one *Nitrosospira* ASV, of which only one ASV was assigned to the species level (*Nitrosomonas oligotropha* –ASV 4354; Fig. 6). There were also many ASVs associated at the family level with *Nitrosomonadaceae*. However, without further metagenomic data, their functional role as AOB cannot be confirmed. Additionally, many of these *Nitrosomonadaceae*-associated ASVs appeared when no or very few AOB were detected by qPCR analysis.

Several *Nitrosospira* ASVs identified by 16S rRNA gene sequencing were exclusively associated with Aquarium 2 bead samples from weeks 1–5 (0.1–0.5% RA), likely corresponding to AOB *amoA* genes that were detected with qPCR for the same samples (Fig. 6). For Aquarium 1, *Nitrosomonas* ASV 1007 was detected for bead and sponge samples for weeks 1, 5, and 9 at less than 0.3% relative abundance, reflecting AOB rarity based on qPCR analysis (Fig. 5). In contrast, there was some discrepancy in the detection of AOB for Aquarium 3 using 16S rRNA gene sequencing and qPCR. Although AOB *amoA* genes were detected in Aquarium 3 with qPCR from weeks 6–12, the *Nitrosomonas* ASV 128 was detected in Aquarium 3 from weeks 7–9 at a relative abundance from 0.8–3.5%. This was higher than the relative abundance of total *Nitrospira* ASVs detected in the same samples at 0.4–1.6%, despite comammox *Nitrospira* dominating over AOB based on qPCR results (Fig. 2, Fig. 6). It is possible that the primers used to detect AOB *amoA* genes were not efficiently targeting the *amoA* sequence associated with this *Nitrosomonas* ASV, which was only found in Aquarium 3.

### Metagenomic analysis of established biofilter microbial communities

Metagenomic sequencing was performed on week 12 samples from each aquarium to further evaluate the presence of nitrification genes. Specific hidden Markov model (HMM) profiles for different groups of nitrifiers were used to search for corresponding reads, alongside a *rpoB* HMM profile to assign overall taxonomy to the unassembled metagenomic reads. Taxonomic profiling of week 12 samples using *rpoB* hits yielded similar results to the community profiles obtained for the same time point with 16S rRNA gene sequencing, yet with increased taxonomic resolution for several ASVs. Genera that were prevalent for all samples from week 12, including *Nitrospira* and *Hyphomicrobium*, were detected in both 16S rRNA gene sequencing and metagenome-based *rpoB* profiling (Fig. 4, Fig. S1). Additionally, *Vicinamibacteraceae* spp. that were unresolved at the genus level using 16S rRNA sequences were present in samples as the genus *Litorilinea* with *rpoB* profiling (Fig. S1). Resolved taxonomy at the genus level for *Caldilineaceae* ASVs was also found with *rpoB* taxonomic profiling, revealing the presence of *Litorilinea* spp. in week 12 samples.

**Figure 7:**
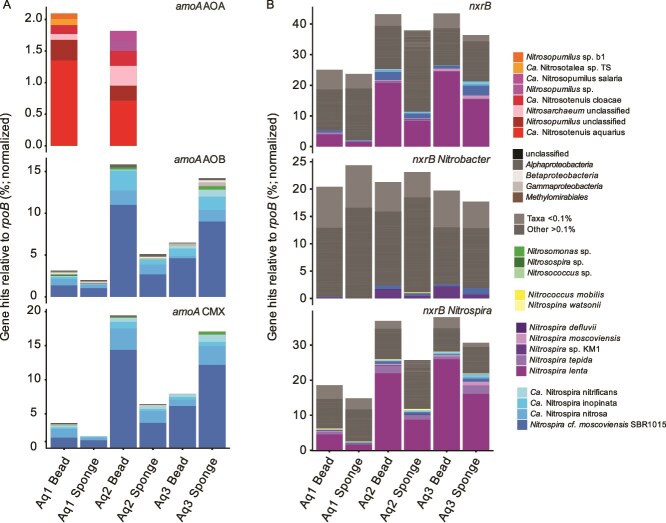
Functional gene profiling of nitrifiers for biofilter bead and sponge samples from week 12 using HMMs. Stacked bars represent the relative abundance (%) of marker genes for nitrification; (A) *amoA* for ammonia oxidation and (B) *nxrB* for nitrite oxidation, with taxonomy for HMM hits assigned to the closest species. All abundances are length-normalized to *rpoB* hits from unassembled metagenomic reads. The specific HMM used for reference is noted on the right-hand side of each bar plot. The *amoA* gene hits are shown with no percent threshold cut-off (A) and *nxrB* gene hits are shown greater or equal to 0.1% (B), normalized to *rpoB*.

The *amoA* HMM profiles reflected those from 16S rRNA gene sequence profiles, detecting AOA in the beads of both Aquarium 1 and 2 (Fig. 6, Fig. 7A). Taxonomic assignment for the majority of AOA *amoA* HMM hits were associated with *Ca.* N. aquarius, consistent with 16S rRNA gene sequencing. The other two taxa detected as hits to both the comammox *Nitrospira* and AOB *amoA* HMM corresponded to both comammox *Nitrospira* and AOB, likely due to sequence homology for the AMO proteins of both groups (Fig. 7). Results revealed that most bacterial ammonia oxidizers in the final bead and sponge samples were associated with comammox *Nitrospira* (~2–14% hits normalized to *rpoB*), with most reads classified specifically to *Nitrospira moscoviensis* SBR1015, which corresponds to a comammox *Nitrospira* MAG (Fig. 7A). For week 12 samples that had detectable AOB using qPCR, HMM hits classified as AOB (including *Nitrosomonas* spp.) were also found for Aquarium 2 bead and sponge samples, and Aquarium 3 sponge at <1% when normalized to *rpoB* genes (Fig. 7A).

The *nxrB* HMM profiles were dominated by *Nitrospira* spp. (Fig. 7B). There were also additional hits to the *nxrB* HMM profiles by nitrate reductase genes, which can be detected by these HMM profiles due to homology between these two enzymes [29]. Read profiles for the *nxrB* HMM showed that most NOB taxa were representative of the canonical NOB *Nitrospira lenta*, with hits for comammox *Nitrospira* showing similar proportions of the same species across samples for *nxrB* HMM hits as were observed for the *amoA* HMMs (Fig. 7A). These results suggest that, alongside comammox *Nitrospira*, there was a substantial proportion of nitrifiers in the biofilters by week 12 that are represented by canonical NOB, which likely also contribute to nitrite oxidation within these systems.

## Discussion

This study shows establishment of all three known groups of autotrophic ammonia oxidizers in freshwater home aquariums and reinforces the dominance of comammox *Nitrospira* in the associated biofilter systems. The results also highlight a need for further research to determine factors influencing microbial community composition and to assess methods for improved nitrifier establishment in biofilters to improve fish health (e.g. microbial inoculation from plant sources, bio-supplements), with further research potentially leading to improved aquarium products that will benefit the aquarium industry. Additionally, the use of aquarium systems to test and evaluate the roles of different aquarium parameters will help to improve the understanding of the microbial ecology in these systems.

Prior aquarium microbiome surveys have focused primarily on fully established biofilters, which show a dominance of AOA [7], or comammox *Nitrospira* [8]. The goal of monitoring microbial communities in freshwater home aquariums during their first 12-weeks was to evaluate how nitrifiers establish within aquarium biofilters. Despite differences in patterns of community establishment, all biofilters from the three independent home aquariums contained similar nitrifiers by the last time point, dominated by comammox *Nitrospira*, which reflects results from recent freshwater aquarium surveys [8], and the current understanding of their niche preferences for slower growth, biofilm, and low ammonia concentrations [20, 30]. When sampling biofilm carrier material from aquarium biofilters, differences were observed in microbial community composition between bead and sponge samples, particularly that AOA preferred beads based on qPCR gene abundances (Fig. 2). This carrier preference has been noted in a previous study where AOA showed a preference for finer sponge substrate than coarser sponge substrate [16]. Using multiple substrates (i.e. surfaces for biofilm growth) may provide distinct niches for growth within biofilters, which may encourage establishment of diverse microbial communities.

When comparing the differences between ammonia oxidizers, faster growth rates of AOB may allow them to increase in abundance early on in response to the higher ammonia concentrations present in the aquarium. However, as the ammonia concentrations become stable, at concentrations below detectable limits, both comammox *Nitrospira* and AOA may outcompete AOB for acquisition of ammonia due to their often-higher ammonia affinities [31, 32]. As a result of this competition, AOB will decrease in abundance as the slower-growing comammox *Nitrospira* and AOA succeed them, becoming the dominant ammonia oxidizers in the biofilter. This succession has been previously observed in similar environments [16], and is reflected in the dominance of both comammox *Nitrospira* and certain AOA species in environments with low ammonia concentrations [8, 11, 29, 33]. With Aquarium 3, there was some succession observed in the biofilter beads, as the AOB *amoA* genes present in the beads decreased below detectable limits in week 11. However, there was limited time to observe these dynamics because of the late establishment of nitrifiers within the biofilter.

We observed a diversity of *nxrB* genes via HMM profiling, despite a lack of direct NOB quantification, from week 12 samples and through the increase in *Nitrospira* ASV diversity in later weeks (Fig. 7, Fig. 6). However, future quantification of NOB during biofilter development would be beneficial to improve our understanding of nitrifier succession in aquarium biofilters. This diversity of *Nitrospira* spp. within biofilters has been observed in other studies [34], although at the time it was unknown that many of these *Nitrospira* could be comammox. The NOB detected in these study aquariums were exclusively *Nitrospira*, which is a highly diverse genera of NOB found ubiquitously in nature, including freshwater and biofilm environments [35]. Other groups of NOB, including *Nitromarin*a, *Nitrospina*, and *Nitrococcus* are more frequently found in marine environments [36, 37], whereas *Nitrotoga* NOB have been found at higher abundance in colder temperature wastewater systems [38]. Overall, the results here are consistent with the niche specialization of different NOB genera.

As observed in similar aquatic biofilm environments, the final communities at 12 weeks contain more than one type of nitrifying species [8, 9], providing beneficial functional redundancy within the biofilter. Nitrifiers also have metabolic versatility, adding flexibility to the substrates they can consume in the event where further competition arises between cohabiting groups. In particular, comammox *Nitrospira* can exhibit varied substrate utilization, because they also possess enzymes to use urea, cyanate, and guanidine [39, 40]. The metabolic versatility of comammox *Nitrospira* has been observed in full-scale wastewater treatment systems, where they actively transcribed urease genes in the system [41]. Much like comammox *Nitrospira*, AOA are also capable of using organic nitrogen substrates, although these capabilities are not present in all AOA species [42]. The potential for mixotrophic activity also exists among AOA, increasing their potential metabolic roles in microbial communities [43–45]. Although the activity of individual ammonia oxidizers was not measured in this study, correlations between nitrification activity alongside the presence and/or absence of nitrifiers was supportive of their activity. Further experiments would be valuable to elucidate the specific roles of each nitrifier group in the aquarium biofilters and determine the direct contributions of each group to ammonia and nitrite oxidation.

In the context of the aquarium biofilter communities, nitrifiers did not develop independently from the other microorganisms and competition for resources including ammonia likely played a role in community dynamics. As noted in previous studies, heterotrophic bacteria can play an important role in nitrifier establishment, assisting with initial biofilm formation in the biofilter [46, 47], which is important for the growth of comammox *Nitrospira* [48]. The phyla observed in the three aquarium biofilters during establishment including dominant *Proteobacteria*, along with *Firmicutes*, *Actinobacteria*, and *Bacteroidetes*, have been observed in freshwater and marine RAS [49]. Additionally, a study observing community succession in a cold freshwater RAS identified several biomarker taxa in the biofilter, including those affiliated with the *Nitrospirales*, *Rhizobiales*, and *Sphingomonadales* [18]. The presence of genera from these orders was also observed in the freshwater aquarium biofilters, with many present at the end of the 12-week sampling period, as detected with taxonomic profiling of metagenomic sequencing data (Fig. S1), suggesting commonality in the taxa observed during biofilter community establishment.

All three independently run aquariums had varied physicochemical parameters. Due to the small sample size and incidental correlations that would result from connecting water chemistry parameters to biofilter microbial community differences between aquariums, it is difficult to draw conclusions on the factors that might have direct influence on the microbial community composition. Ideally, conducting further controlled experiments with a larger sample size could help to distinguish among these effects. Previous literature demonstrates that nitrifiers vary in their environmental preferences [50, 51]. Because only freshwater aquarium biofilters were examined in this study, the detected nitrifiers had a niche preference for freshwater environments. Although some comammox *Nitrospira* spp. can adapt to higher salinity [52], they have yet to be detected in marine environments, including saltwater aquarium biofilters [8]. This made the inclusion of saltwater aquarium samples less relevant when assessing the presence of ammonia oxidizers during microbial community establishment in aquarium biofilters, because the current understanding is that comammox *Nitrospira* are absent from marine aquariums. Previous research has explored microbial community succession and effects of perturbation in experimental saltwater aquariums, specifically with only two independently established aquariums [21]. They indicated that changes in microbial community patterns were not always correlated with water chemistry measurements and replicable patterns of community succession in both aquariums. The saltwater aquarium study indicated that more targeted experimental approaches to understand the specific impacts of changing conditions on microbial community succession and stability.

Although it was challenging to elucidate factors responsible for the different microbial community compositions observed in the biofilters, differences in nitrifiers among the three aquariums raise several questions: Why did AOB establish in Aquarium 2 but not in Aquarium 1? Why was nitrification activity delayed in Aquarium 3? Based on small measured increases in detectable nitrite and nitrate in Aquarium 3 from week 1–6, low levels of nitrification were likely occurring; however, consistent nitrification activity was not observed until week 8. Despite some aquarium-specific variation, it is clear from the water chemistry data that nitrification kept ammonia levels below detectable limits by the end of the 12-week sampling period in all three aquariums. There was one apparent difference between the aquariums with and without early nitrification, which was the inclusion of live plants in both Aquariums 1 and 2, but not in Aquarium 3. We hypothesize that live plants served as a source of microorganisms, introducing nitrifiers that may have decreased the time required to establish an effective nitrifying community. Many rhizosphere and phyllosphere microorganisms would be transferred into the new aquarium upon addition of the plants. In aquaponics systems, nitrifying bacteria are often present in the system and sometimes associated with plant roots, including comammox *Nitrospira* and AOA [53, 54]. Elevated 16S rRNA gene copies per bead and sponge sample detected in the pre-fish sampling for Aquariums 1 and 2 support the hypothesis that they had a higher initial inoculation of microorganisms prior to the addition of fish, which might have aided in microbial community establishment. In addition to a potential microbial inoculation source, the live plants in Aquariums 1 and 2 provided some nitrate removal from the water via assimilatory processes, possibly explaining lower average nitrate concentrations for Aquarium 1 (4.2 ± 0.1 mg/L NO_3_^-^-N) and Aquarium 2 (7.2 ± 1.3 mg/L NO_3_^-^-N) compared to Aquarium 3 (9.5 ± 1.5 mg/L NO_3_^-^-N) across weeks 9–12.

Results from this study have quantified the abundances of different ammonia oxidizers within the biofilters, coinciding with observed nitrification activity through ammonia, nitrite, and nitrate concentration measurements. Although these concentrations were measured during the 12 weeks of aquarium sampling, the amount of nitrogen added to the aquarium system (e.g. via fish food) was not quantified, nor is it known the specific amount of nitrogen assimilated by plants in Aquariums 1 and 2. As a result, it was not possible to determine a specific nitrification rate for these aquariums. However, by comparing changes in ammonia, nitrite, and nitrate concentrations over the 12 weeks, it could be determined when nitrification activity began. Additionally, high final nitrate concentrations, ranging from ~5–10 mg/L NO_3_^-^-N further indicates that nitrogen coming into the aquarium system was cycled through nitrification to nitrate, with nitrification activity once established, able to keep ammonia and nitrite concentrations below detectable limits. With a better sense of ammonia oxidizer presence in the biofilters during the first 12-weeks of microbial community establishment, future studies quantifying nitrification rates and activity of specific nitrifiers within the biofilters would further clarify their contributions to nitrification in these aquariums.

Aquarium hobbyists seek to establish nitrifying communities within new home aquarium biofilters, to maintain healthy water conditions for their aquatic residents (i.e. fish). Biological supplements commercially available in pet supply stores typically contain AOB and NOB [7, 55], which are meant to accelerate nitrifier establishment in the biofilter and help keep ammonia levels low. In this study the sole aquarium that added supplements during set up (i.e. Aquarium 3) was not associated with nitrite or nitrate accumulation until week 6–8 and known nitrifiers were also not detected in biofilter DNA until that time. As discussed, other factors may have delayed nitrification activity, which are not apparent given the present data. However, with a sample size of one aquarium, it is not possible to conclude that these supplements would be ineffective when used as instructed in another aquarium. It is known that supplementation in the form of either adding in filter material from a previously established biofilter or other forms of inoculation have been effective ways to promote nitrifier growth in biofilters [13, 56]. However, a recent study revealed that only one of five tested quick-start nitrification products sold with the purpose of promoting rapid aquarium nitrification significantly reduced aquarium ammonia concentrations [57]. The effective quick start nitrification product claims to contain *Nitrosomonas*, along with *Nitrospira*, although this likely refers to canonical NOB. The results from this home aquarium establishment study draw further attention to the high abundance of comammox *Nitrospira* in freshwater aquarium biofilters, which occurs in tandem with detectable nitrification activity in the aquariums. These results suggest that aquarium bio-supplements would benefit from including comammox *Nitrospira* to promote nitrification activity in home aquarium biofilters and a sustainable nitrifying community.

Establishment of nitrifiers in Aquariums 1 and 2 without supplementation demonstrates that nitrifiers can be seeded in aquarium biofilters without the use of bio-supplements. Shared *Nitrospira* and AOA ASVs in these biofilters suggests that these nitrifiers could originate from the municipal drinking water system. Comammox *Nitrospira* have been detected within drinking water systems previously [14, 58], in addition to both AOA and AOB also residing in drinking water distribution systems [59–61]. Because participants were provided with gift cards to obtain fish, live plants, and supplies from the same local pet store, the nitrifiers may have originated from this common source and transferred by the fish, plants, and water associated with their purchase.

In the future, long-term monitoring of aquarium biofilter systems past twelve weeks would confirm comammox *Nitrospira* stability in the biofilter community, which has been reported before for AOA in aquarium biofilters [16]. Overall, results from this study have highlighted the previously overlooked presence of comammox *Nitrospira* during the establishment of nitrifying microbial communities in freshwater aquarium biofilters. This suggests a need for future studies to investigate the individual contributions of ammonia and nitrite oxidizers to nitrification in these biofilters during their development.

## Supplementary Material

McKnightms_supplemental_ycaf212(1)

ASV_table_ycaf212

## Data Availability

All raw sequence reads associated with both the metagenomic and 16S rRNA gene sequencing were deposited in the NCBI Sequence Read Archive under BioProject accession number PRJNA1162135.
