# Supplementary material for: Microbial community succession of home aquarium biofilters associated with early establishment of comammox Nitrospira
Source: ISME Commun. 2025 Nov 14;5(1):ycaf212. doi: 10.1093/ismeco/ycaf212 (PMC12704419; doi:10.1093/ismeco/ycaf212)
Supplement: McKnightms_supplemental_ycaf212(1) [file mcknightms_supplemental_ycaf212(1).docx]

**Supplemental material**

**Microbial community succession of home aquarium biofilters associated with early establishment of comammox *Nitrospira***

Michelle M. McKnight^1^, Natasha Szabolcs^1^, Alyssa K. Graham^1^, Josh D. Neufeld^1*^

^1^Department of Biology, University of Waterloo, Waterloo, Ontario, Canada, N2L 3G1

*Corresponding author: Department of Biology, University of Waterloo, 200 University Avenue West, Waterloo, Ontario, N2L 3G1, Canada. Tel. +1 519-888-4567; Fax +1 519-746-0614 E-mail: jneufeld@uwaterloo.ca

**Keywords:** Aquarium, biofilter, nitrification, 16S rRNA gene sequencing, microbial community succession, metagenomics

Supplemental methods

*Home aquarium set-up and sample collection*

Each of the three participants received a 20-gallon aquarium, an LED light lid, an AquaClear 30 aquarium filter, and a plastic thermometer (Hagen Industries). Each aquarium was established with independent selections of fish, fish food, aquarium treatment products, accessories, and operational conditions (e.g., temperature, water source) (Table 1). Aquarium 3 was the only aquarium in this experiment to which Cycle (Hagen Industries), reported previously to contain AOB and NOB [1], was added during set-up; the other two aquariums had no supplements added. Participants were free to establish an aquarium to their liking to represent realistic and unique home aquarium conditions.

Participants regularly sampled ceramic beads, sponge, and water from their tanks as instructed. Samples were first collected 24 hours prior to the addition of fish, 48 hours after adding fish, then weekly thereafter for 12 weeks. During each sampling event, three biofilter beads were collected using flame-sterilized tweezers and sponge samples of approximately 2.5 x 1 x 1 cm^3^ in size were collected with flame-sterilized scissors. Both bead and sponge samples were collected and stored in sterile 1.5-mL tubes. Water samples were collected in 50-mL tubes. All samples were stored after collection at -20°C until processed.

*DNA extraction*

A 1 x 1 x 2 cm^3^ piece of each sponge sample was cut into smaller pieces using flame sterilized scissors and tweezers and placed into PowerBead Pro Tubes (Qiagen). After adding lysis solution, tubes were incubated at 65°C for 30 min, followed by bead beating at 5.5 m/s for 45 s using a FastPrep-24 bead beater (MP Biomedical, Santa Ana, CA). After sample lysis, the remainder of the DNA extraction procedure was completed as described by the manufacturer’s protocol. For DNA extraction from the ceramic beads, beating beads from the PowerBead Pro Tubes were emptied into sterile 5 mL centrifuge tubes (Globe Scientific Inc.). One ceramic bead was placed into the 5 mL tube using flame sterilized tweezers, along with 800 μL lysis solution (Solution C1, Qiagen). The tubes were then incubated at 65°C for 10 min prior to bead beating, which was performed by attaching tubes to an Analog Vortex Mixer (ThermoFisher Scientific) run at maximum speed (3200 rpm) for 10 min. Tubes were centrifuged for 1 min at 7100 RCF before continuing the extraction following the manufacturer’s protocol. Extracted DNA was eluted to a final volume of 100 μL using the kit elution buffer (Solution C6, Qiagen). Subsequently, to assess the quality of extracted DNA, samples were run on a 1% agarose gel stained with EtBr.

*Quantitative PCR*

Each qPCR contained 5 μL of 2X SsoAdvanced Universal SYBR Green Supermix (Bio-Rad, Hercules, CA, USA), 3 pmoles of both forward and reverse primer, 5 μg of bovine serum albumin, and 1-10 ng of template DNA topped up to a final reaction volume of 10 μL with PCR water. All samples were run as technical duplicates, including standards and no template control samples. Amplifications conditions for the 515F-Y/806R primer set included an initial denaturation at 98°C for 3 min, followed by 40 cycles of 98°C for 30 s, 50°C for 15 s, and 72°C for 30 s. A melt curve was run immediately following amplification from 65-95°C, increasing at intervals of 0.5°C for 2 s each. Standard curves were generated for each qPCR run using purified template DNA containing the full-length 16S rRNA gene of *Thermus thermophilus*, with copy numbers ranging from 10^1^-10^8^ copies/μL.

*16S rRNA gene sequencing*

Demultiplexing of the paired-end sequence reads was carried out using MiSeq Reporter software version 2.5.0.5 (Illumina). The AXIOME3 (Automation, eXtension, and Integration Of Microbial Ecology) pipeline, implementing QIIME2 version 2020.6 and DADA2, was used for primer sequence removal, quality trimming, denoising, paired-end sequence merging, and subsequent generation of an amplicon sequence variant (ASV) table [2, 3]. Taxonomic classification of ASVs was performed during the AXIOME3 pipeline using the SILVA database release 138 [4], along with generation of a phylogenetic tree of ASV sequences using FastTree [5]. Beta diversity metrics were calculated using the q2-diversity plugin also via the AXIOME3 pipeline [3]. Additional statistical analysis of beta diversity was performed in R using the adonis2 function from the vegan package (v. 2.6-4) to test for differences in beta diversity between sample groups (i.e., aquarium and filter material type). The pairwise.adonis function from the pairwiseAdonis R package (v. 0.4.1) was subsequently used to test for specific differences in beta diversity between individual groups, using Bonferroni correction of *p* values to account for multiple comparisons. Calculation of alpha diversity indices, including observed species (richness), Pielou’s index (evenness), and Shannon index (richness and evenness), was done using the vegan package (v. 2.6-4) implemented in R (v. 4.3.2).

*Metagenomic sequencing and analysis*

Extracted DNA was sent to The Centre for Applied Genomics (TCAG) in Toronto, ON, CA. For each aquarium sponge sample, 700 ng of DNA was supplied for sequencing, whereas a minimum of 140 ng of DNA was sent for bead samples because they had a lower yield of extracted DNA. Sequencing libraries were prepared using the TruSeq DNA PCR-Free HT Library Prep Kit (Illumina) following the manufacturer’s protocol. Prepared libraries were then sequenced on an NovaSeq 6000 System using a single SP flow-cell lane (2×150 cycles) (Illumina). Demultiplexed sequence data received from TCAG were processed using the quality control portion of the Metagenome ALTAS pipeline to remove duplicates, perform quality trimming, and contaminant removal (i.e., adapter sequences) [6]. Subsequently, FragGeneScanRs (v 1.1.0) was used to predict open reading frames (ORFs) within the quality controlled, unassembled, and unmerged forward and reverse metagenomic reads [7]. Hidden Markov model (HMM) profiles for associated functional genes of interest involved in nitrification (AOA_*amoA*, AOB_*amoA*, comammox_*amoA*, *nxrB*, Nitrobacter_*nxrB*, and Nitrospira_*nxrB*) were retrieved, along with the *rpoB* HMM for taxonomic classification from the FunGene database [8]. These HMM profiles were used with the MetAnnotate (metannotate-wrapper; v. 0.9.2) tool to quantify the relative abundances and taxonomic affiliations of sequence hits to functional marker genes from the unassembled metagenomic reads [9]. The NCBI RefSeq release 220 (October 2023) was used for taxonomic assignment within MetAnnotate using the default E-value (1×10^-6^) for usearch-based taxonomic classification. MetAnnotate results were further analyzed and visualized using the MetAnnoviz R package (v. 1.1.0), which normalized functional gene hits for each HMM based on HMM length and the number of length-normalized hits to the *rpoB* HMM. An E-value of 1×10^-10^ was used as the threshold above which hits to HMMs would not be plotted.

**References**

1. Sauder LA et al. Aquarium nitrification revisited: *Thaumarchaeota* are the dominant ammonia oxidizers in freshwater aquarium biofilters. *PLoS One* 2011;**6**:e23281. https://doi.org/10.1371/journal.pone.0023281

2. Callahan BJ et al. DADA2: High resolution sample inference from Illumina amplicon data. *Nat Methods* 2016;**13**:581. https://doi.org/10.1038/NMETH.3869

3. Min D, Doxey AC, Neufeld JD. AXIOME3: Automation, eXtension, and integration of microbial ecology. *Gigascience* 2021;**10**:gigab006. https://doi.org/10.1093/gigascience/giab006

4. Quast C et al. The SILVA ribosomal RNA gene database project: improved data processing and web-based tools. *Nucleic Acids Res* 2013;**41**:D590–D596. https://doi.org/10.1093/NAR/GKS1219

5. Price MN, Dehal PS, Arkin AP. FastTree 2 – approximately maximum-likelihood trees for large alignments. *PLoS One* 2010;**5**:e9490. https://doi.org/10.1371/JOURNAL.PONE.0009490

6. Kieser S et al. ATLAS: A Snakemake workflow for assembly, annotation, and genomic binning of metagenome sequence data. *BMC Bioinform* 2020;**21**:257. https://doi.org/10.1186/s12859-020-03585-4

7. Van der Jeugt F, Dawyndt P, Mesuere B. FragGeneScanRs: faster gene prediction for short reads. *BMC Bioinform* 2022;**23**:198. https://doi.org/10.1186/s12859-022-04736-5

8. Fish JA et al. FunGene: The functional gene pipeline and repository. *Front Microbiol* 2013;**4**:291. https://doi.org/10.3389/fmicb.2013.00291

9. Petrenko P et al. MetAnnotate: Function-specific taxonomic profiling and comparison of metagenomes. *BMC Biol* 2015;**13**:92. https://doi.org/10.1186/s12915-015-0195-4

Table S1 Quantity of DNA extracted from bead and sponge samples and water chemistry concentrations. Date column indicates the specific day of sampling. For ammonia, concentrations denote the total ammonia present in the sample, including both ionized and unionized forms. Samples noting <5 ng of DNA were below the detection limit for the Qubit instrument used for DNA quantification.

|  | Sample | Date | Bead DNA (ng) | Sponge DNA (ng) |
| --- | --- | --- | --- | --- |
| Aquarium 1 | Pre-fish | 09/02 | 255 | 241 |
|  | Post-fish | 09/03 | 28 | 44 |
|  | Week 1 | 09/12 | 165 | 132 |
|  | Week 2 | 09/19 | 155 | 161 |
|  | Week 3 | 09/28 | 242 | 652 |
|  | Week 4 | 10/03 | 425 | 945 |
|  | Week 5 | 10/10 | 933 | 5900 |
|  | Week 6 | 10/20 | 1050 | 3140 |
|  | Week 7 | 10/25 | 640 | 1640 |
|  | Week 8 | 10/31 | 288 | 3900 |
|  | Week 9 | 11/07 | 326 | 1560 |
|  | Week 10 | 11/14 | 307 | 2470 |
|  | Week 11 | 11/21 | 356 | 2250 |
|  | Week 12 | 12/01 | 344 | 1780 |
| Aquarium 2 | Pre-fish | 09/03 | 512 | 1130 |
|  | Post-fish | 09/05 | 179 | 647 |
|  | Week 1 | 09/12 | 213 | 120 |
|  | Week 2 | 09/19 | 120 | 144 |
|  | Week 3 | 09/26 | 241 | 210 |
|  | Week 4 | 10/03 | 263 | 385 |
|  | Week 5 | 10/10 | 1210 | 16810 |
|  | Week 6 | 10/17 | 604 | 17770 |
|  | Week 7 | 10/24 | 1460 | 9780 |
|  | Week 8 | 10/31 | 1990 | 15390 |
|  | Week 9 | 11/07 | 1280 | 13950 |
|  | Week 10 | 11/14 | 2240 | 15730 |
|  | Week 11 | 11/21 | 2220 | 13800 |
|  | Week 12 | 12/05 | 5920 | 23120 |
| Aquarium 3 | Pre-fish | 09/07 | <5 ng | <5 ng |
|  | Post-fish | 09/09 | 1070 | 915 |
|  | Week 1 | 09/17 | 456 | 228 |
|  | Week 2 | 09/24 | 578 | 970 |
|  | Week 3 | 10/01 | 224 | 174 |
|  | Week 4 | 10/08 | 277 | 395 |
|  | Week 5 | 10/15 | 303 | 3850 |
|  | Week 6 | 10/22 | 681 | 2480 |
|  | Week 7 | 10/29 | 1580 | 4420 |
|  | Week 8 | 11/05 | 1610 | 4900 |
|  | Week 9 | 11/12 | 1550 | 8920 |
|  | Week 10 | 11/19 | 414 | 8100 |
|  | Week 11 | 11/26 | 687 | 10140 |
|  | Week 12 | 12/03 | 480 | 9920 |


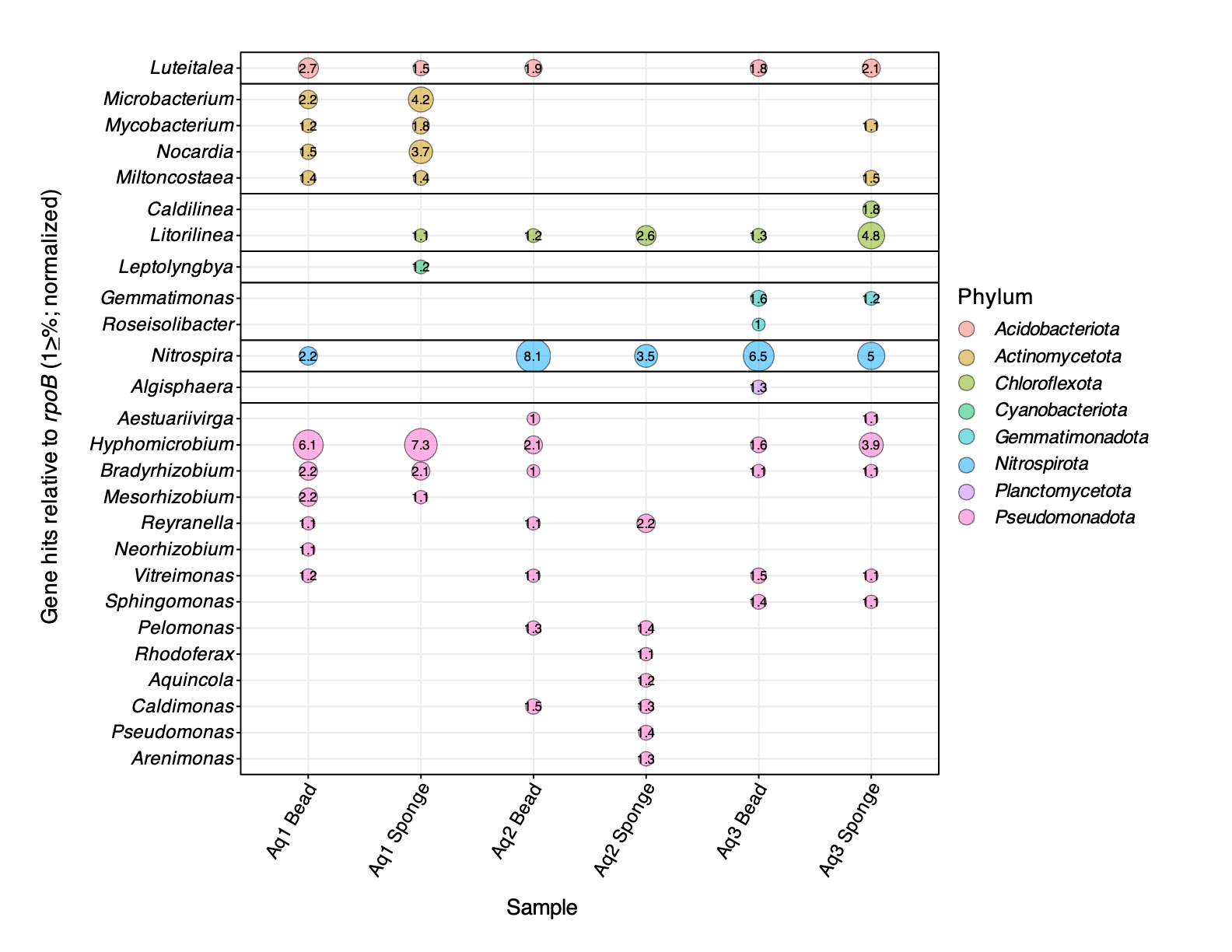


Figure S1 Genera of microorganisms detected using the taxonomic marker gene *rpoB* HMM in week 12 bead and sponge samples from metagenomic sequencing. Only genera that were greater than or equal to 1% relative abundance within a sample are shown in the bubble plot, where bubbles and number on plot correspond to the relative abundance of each genera.
